# Looped Audiovisual Health Education Talk Reduces Vaccine Clinic Wait Time in Nigeria

**DOI:** 10.1111/cch.70152

**Published:** 2025-08-04

**Authors:** Rosena O. Oluwafemi, Bukola Ajayi, Eneida A. Mendonca, Paul Biondich, Osayame A. Ekhaguere

**Affiliations:** ^1^ Department of Pediatrics Mother and Child Hospital Akure Ondo State Nigeria; ^2^ Ondo State Ministry of Health Akure Nigeria; ^3^ Division of Bioinformatics, Cincinnati Children's Hospital, Departments of Pediatrics and Biomedical Informatics University of Cincinnati Cincinnati USA; ^4^ Department of Pediatrics Indiana University School of Medicine Indianapolis Indiana USA; ^5^ Regenstrief Institute, Inc. Indianapolis Indiana USA

**Keywords:** clinic wait time, health education, low‐ and middle‐income countries, routine childhood vaccines, vaccine health talk, vaccines

## Abstract

**Background:**

Wait time at vaccine clinics is a barrier to routine childhood vaccinations in low‐ and middle‐income countries (LMICs). Waiting for a critical mass of clients to accrue before conducting the vaccine health education talk prolongs clinic time.

**Methods:**

We implemented a workflow change, including a looped audiovisual vaccine education talk on a solar‐powered television. We compared clients' average clinic time using a before‐and‐after study, time‐motion design, and surveyed providers and clients on their perspectives on the workflow change.

**Results:**

In the post‐implementation phase, compared to the pre‐implementation phase, the average clinic time for all clients and the subgroup who presented before 9:00 AM was significantly reduced by 13 and 31 min, respectively (*p* = 0.006 and < 0.000). Providers and clients were positive about the workflow change.

**Conclusion:**

A looped audiovisual vaccine education talk significantly reduces client vaccine clinic wait time and is acceptable to providers. Research on the impact of workflow change with alternate vaccine health talk delivery mode on vaccine uptake and completion is required.

## Summary

- The long wait time clients experience during routine childhood vaccine clinic sessions in low‐ and middle‐income countries is a reported barrier to childhood vaccine uptake and completion.
- Standard workflow of waiting for a critical mass of clients to accumulate before delivering the group vaccine health education talk is a reported key driver of long wait times.
- Implementing a workflow change that included looped audiovisual vaccine education talk at routine childhood vaccine clinics in Nigeria was feasible and significantly reduced client vaccine clinic wait time.
- The workflow change utilizing a looped audiovisual vaccine education talk at routine childhood vaccine clinics in low‐ and middle‐income countries was acceptable to providers and clients.

## 1 Introduction

The barriers to routine childhood vaccine uptake and completion are complex (Kaufman et al. 2021). Appropriate vaccine literacy is critical to the parental acceptance of vaccines for their child (Kaufman et al. 2021; Willis et al. 2013; Kaufman et al. 2017). In low‐ and middle‐income countries (LMICs), vaccine clinic health talks are the primary source of vaccine education (Oku et al. 2016; Ames et al. 2015). Vaccine health education talks occur in real‐time and often precede the administration of vaccines (Oku et al. 2016; Ames et al. 2015; Ekhaguere et al. 2021). Evidence suggests that providers delay the start time of vaccine administration to garner a critical mass of clients before providing vaccine education and health talks (Ekhaguere et al. 2021). This workflow prolongs total clinic wait time and is a barrier to vaccine uptake and completion, as reported by clients (Oku et al. 2016; Ames et al. 2015; Ekhaguere et al. 2021; Weiner et al. 2017; Acceptability of Intervention Measure (AIM) n.d.). One study found clients deliberately arrive late to the clinic—missing the health education session altogether—to avoid the health education talk and cut down wait time (Ekhaguere et al. 2021). The current study aimed to assess the impact of an alternate vaccine clinic workflow that substituted a looped audiovisual health education talk for the talks that occur in real‐time.

## 2 Methods

### 2.1 Study Design and Setting

We conducted a before‐and‐after time‐motion observational study to pragmatically assess the immediate effects of the workflow change on clinic wait time in a real‐world setting, and a descriptive qualitative study utilizing structured surveys to explore provider and parental perceptions of the study intervention. The study was conducted at Mother and Child Hospital, Akure, Ondo State, Nigeria, between August 2022 and October 2022. Ondo State is in the southwest region of Nigeria. The main local language is Yoruba.

The proportion of children receiving the third diphtheria‐tetanus‐pertussis (DPT3) containing vaccine—the global childhood vaccine benchmark—in Ondo State, Nigeria, is 63%, but regional disparities exist (Ekhaguere et al. 2019).

The Mother and Child Hospital is a state‐run referral hospital that provides free maternal and child healthcare services, primarily to low‐ and middle‐income families. The vaccine clinic is housed within the hospital but operates under the supervision of the State Primary Health Care Board and the Ministry of Health.

#### 2.1.1 Study Participants and Sampling

Two participant groups were included: (1) health providers involved in clinic operations—community health extension workers, certified nurses, public health workers and clinic directors—and (2) clients (parents or caregivers) who brought a child for vaccination.

We used a convenience sampling strategy to recruit providers and clients. For the observational time‐motion component, we recorded the arrival and discharge times of all clients who attended the clinic on the pre‐ and post‐intervention study days. For the staff survey, we included all health providers on duty at the immunization clinic on the designated study day. For the client survey, we included only those who had visited the clinic at least once prior to the intervention, allowing them to compare their experience before and after the workflow change.

#### 2.1.2 Study Recruitment

At the beginning of the clinic session, the study research staff approached potential survey participants and explained the purpose and procedure of the study. Due to their extensive involvement in the study activity, providers gave written informed consent. Clients gave only verbal consent, as their participation was limited to answering four survey questions about their perception of clinic time and workflow changes.

Since we only observed clinic admission and discharge times for clients, no specific recruitment was required or performed for the observational time–motion component.

The study research staff were public health professionals trained in health systems research and survey methodology. Research assistants were trained to administer surveys ethically and neutrally in both English and Yoruba. They were fluent in the local language and familiar with the study setting, which helped minimize social desirability bias and power differentials during data collection. Interviewers had no prior relationships with participants.

### 2.2 Study Intervention and Procedure

The intervention consisted of replacing the live vaccine health talk with a pre‐recorded 22‐min health talk video, delivered via a solar‐powered television installed in the clinic. The video content was developed through an iterative process by the clinic staff and reviewed by a representative from the Ondo State Ministry of Health. The first half of the video was in English, and the second half was in Yoruba. The video played on a loop, with a 10‐min question and answer session between each playback.

The general pre‐implementation vaccine clinic workflow is summarized as follows: (1) The clinic opens to clients at 8:00 AM, and clients begin to assemble; (2) at client arrival, providers locate their duplicate vaccine cards kept at the clinic; (3) providers complete the vaccine card, clinic and government reporting documentation; (4) providers obtain and record the anthropometric measurements of the client; (5) vaccine talk is performed; (6) vaccines are administered; and (7) clients are discharged from the clinic. The vaccine talk would typically occur between 9:30 and 10:00 AM. Clients who arrived after the vaccine talk missed the education on vaccines.

The modified post‐implementation vaccine clinic workflow is summarized as follows: (1) The clinic opens to clients at 8:00 AM, and clients begin to assemble; (2) vaccine talk is played on a loop starting at 8:00 AM for 22 min with 10 min for questions. Clients who were present and listened to the vaccine talk then complete Steps 3 to 7, as outlined above. While Steps 3 to 7 are going on, new clients who arrive get the opportunity to listen to the next loop of vaccine talk and then complete Steps 3 to 7 afterwards. This system continues until no new clients arrive.

The provider survey was conducted at the end of the clinic session, and the client survey was conducted after discharge from the clinic.

### 2.3 Data Collection Analysis

#### 2.3.1 Quantitative Component

To evaluate the impact of the study intervention on clinic time, we recorded arrival and discharge times of all clients on one clinic day before and one clinic day after the implementation of the intervention. Total clinic time was calculated for each client, and an unpaired Student's *t*‐test was used to compare mean visit duration across the two time points. We also performed a post hoc analysis on a subgroup of clients who arrived before 9:00 AM, as published data suggest that clients who arrive before 9:00 AM experience the longest clinic time (Ekhaguere et al. 2021).

#### 2.3.2 Qualitative Component

We administered structured surveys to assess providers and clients' perception of the study intervention. We utilized a 3‐point Likert scale to assess clients' perception of (1) time spent during the intervention visit, (2) time spent in clinic compared to previous visits, (3) perception of the educational content of the recorded health talks and (4) the adequacy of time allowed for questions. There was also one open‐ended question for suggestions on improving the new workflow. All client surveys were interviewer‐administered to mitigate literacy barriers.

The provider survey assessed the acceptability of the workflow change and was adapted from a validated instrument with established psychometric properties (Bangure et al. 2015; Bosu et al. 1997). Provider surveys were self‐administered. Surveys took approximately 5 min (caregivers) and 10 min (providers) to complete. The survey instruments were pretested with a similar population to ensure clarity and appropriateness.

Qualitative data from open‐ended responses were analysed using basic content analysis to identify recurring suggestions and feedback themes. Two study team members reviewed responses independently and reached consensus through discussion. The short and targeted nature of the open‐ended questions did not necessitate full thematic analysis.

### 2.4 Ethical Consideration

The study was approved by the Ondo State Ministry of Health (NHREC/18/08/2016). Each provider recruited for the survey provided signed informed consent, and each client surveyed provided verbal consent.

## 3 Results

**FIGURE 1:**
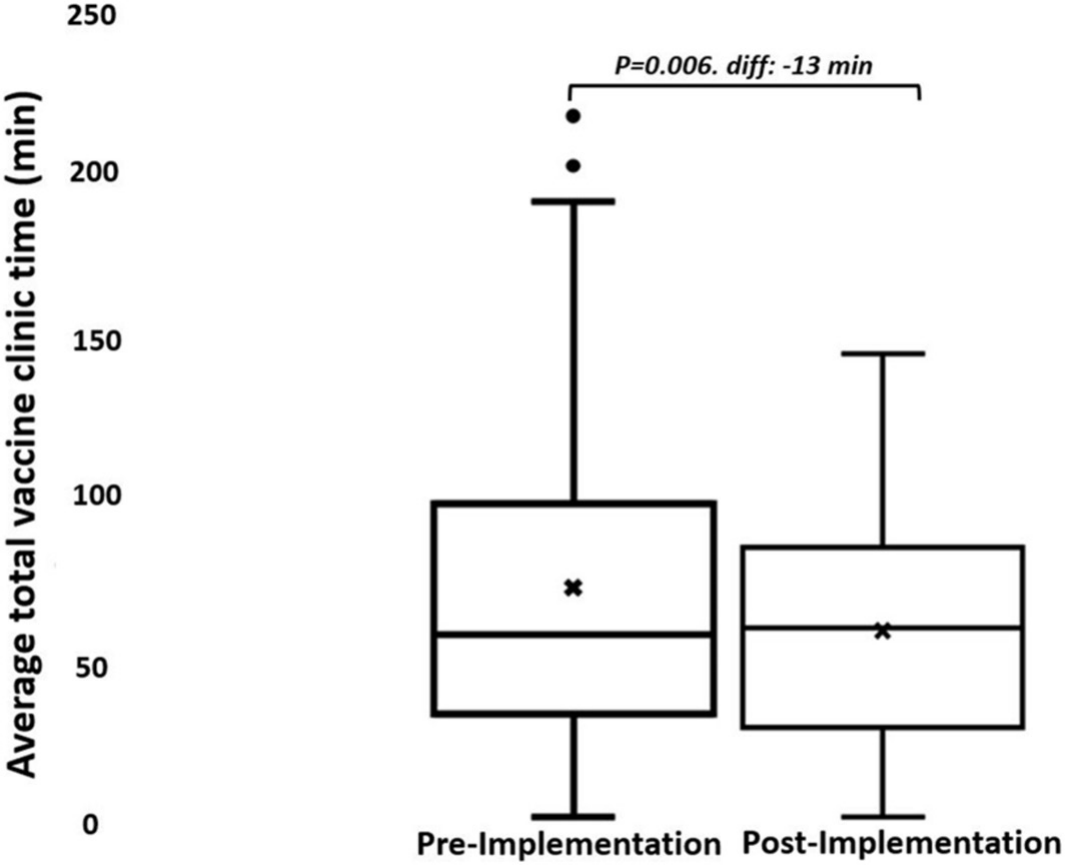
Average total clinic time for clients.

We collected clinic time data from 224 and 145 clients in the pre‐ and post‐implementation phases, respectively. In the pre‐implementation phase, the longest clinic time was 215 min, and the shortest was 3 min. In the post‐implementation phase, the longest clinic time was 143 min, and the shortest was 3 min. The average pre‐ and post‐implementation total clinic times were 72 (SD, 49) and 59 (SD 35) minutes, respectively (*p* = 0.006) (Figure 1).

**FIGURE 2:**
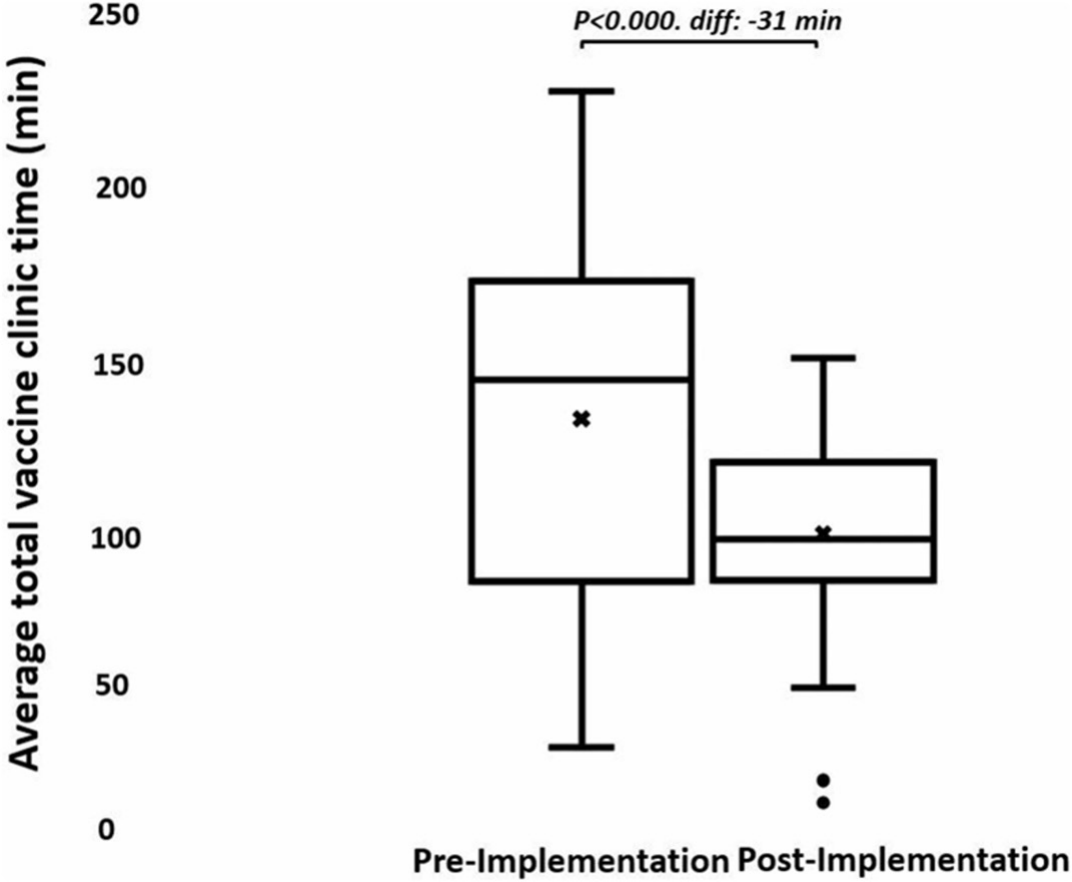
Average total clinic time for clients arriving at or after 9 AM.

In the post hoc analysis, 23% of the pre‐implementation group arrived before 9:00 AM, and 31% of the post‐implementation group arrived before 9:00 AM. Of those who arrived before 9:00 AM, the average pre‐ and post‐implementation total clinic times were 127 (SD, 46) and 95 (SD 26) minutes, respectively (*p* = < 0.000) (Figure 2).

We surveyed 100 clients who had visited the clinic at least once before implementing the workflow change. The workflow changes positively impacted most clients (Table 1). Concerning post‐implementation time in the clinic, 53% felt it was better, 39% the same and 8% reported it was worse than the pre‐implementation phase. A minority of clients (26%) thought the time in the clinic was too short; the rest felt it was either adequate (50%) or still too long (24%). All the clients felt there was adequate time for questions and that the video provided better or the same vaccine health education.

**TABLE 1:** Client perception of the length of time spent in the vaccine.

Question	Responses (*N* = 200)		
Time in clinic today, *n* (%)	Too short 52 (26)	Adequate 100 (50)	Too long 48 (24)
Time in clinic today compared to last visit, *n* (%)	Worse 16 (8)	Same 78 (39)	Better 106 (53)
Video provided same or better health education, *n* (%)	Disagree 0 (0)	Neutral 4 (2)	Agree 196 (98)
Adequate time to ask questions, *n* (%)	Disagree 0 (0)	Neutral 2 (1)	Agree 198 (99)

The demographic information of the providers (13) is provided in Table 2. All acceptability questions were answered as either agree or completely agree. None of the providers felt their workflow was negatively impacted by implementing the new vaccine talk delivery method. The themes from the written comments from providers include the following:

- *Improved work efficiency*: One provider wrote, ‘The project has greatly improved the immunization services at the center’.
- *Better client flow*: One wrote about the new workflow: ‘It has reduced the stress and time spent giving health talks’.
- *Reduction in repetition*: One provider wrote, ‘It has reduced patients' waiting time and prevents us from giving repeated health talks’.
- *Enhanced service quality*: One provider wrote, ‘The project has greatly improved the immunization services at the center’.

**TABLE 2:** Clinic provider demographic information.

Characteristics	
Age in years, median, IQR	45 (42–52)
Years in service, median, IQR	15 (13–22)
Years working in study clinic	7 (7–12)
Highest degree, *n* (%)	
• Secondary school leaving certificate	1 (8)
• Ordinary national diploma	8 (61)
• Higher national diploma	1 (8)
• Bachelor's degree	2 (15)
• Master's degree	1 (8)

## 4 Discussion

We evaluated the impact of a vaccine clinic workflow change on the average clinic time for clients in a vaccine clinic in southwest Nigeria. The essential component of the workflow change was a video‐recorded vaccine education health talk played on a loop rather than the traditional in‐person talk by clinic staff. The workflow changes significantly reduced the average clinic time and the time for a subset of clients who presented before 9:00 AM. While not universal, most clients visiting the clinic responded positively to the workflow change and vaccine talk delivery mode. Providers accepted the workflow change and felt it positively impacted their workflow.

Long clinic wait times have been documented as barriers to vaccinations, especially in LMICs. Unlike high‐income countries, most vaccine clinics in LMICs do not set specific appointment time slots for clients (Jani et al. 2008). Patients are expected to gather, and vaccines are administered on a first‐come, first‐served basis after completing key activities. Waiting for a critical mass of patients to accumulate before delivering the vaccine talk has been shown to be a significant source of lengthy clinic wait times (Ekhaguere et al. 2021). Our study is the first to test an audiovisual vaccine talk on vaccine clinic wait time in a group vaccine clinic setting in a LMIC. We demonstrate that overall, and particularly for those who arrive early (before 9:00 AM), the client's wait time was significantly reduced compared to the standard format.

The unique workflow of public immunization clinics in Nigeria differs from the one‐on‐one provider‐patient interaction common in high‐income countries (Kaufman et al. 2018), likely leading to the dearth of published research on using audiovisuals to reduce clinic time. However, clinic‐based audiovisual education has been utilized for other vaccine‐related interventions. It has been shown to improve vaccine uptake (Tefera et al. 2018) and enhance parents' ability to manage vaccine‐related side effects in high‐income countries.

One consequence of the long wait time dictated by the vaccine talk delivery time is that clients begin to ‘game’ the system and deliberately present late, after the expected time for the vaccine talk; consequently, they miss out on crucial vaccine health information. A looped video vaccine talk prevents clients from missing the health talk while minimizing the overall clinic wait time. In the current study, 76% of the clients surveyed felt the clinic time was either adequate or too short. However, only 53% felt it was better compared to the pre‐implementation phase. One possible reason for this average‐rated perception is that the study was performed during the week of implementation, when the workflow had not been fully normalized. Additionally, some clients have had other appointments for themselves or their child. We did not investigate this potential conflict.

This study was a feasibility study that proved that modifying the clinic workflow could significantly reduce the clinic wait time. One key limitation of this study is its generalizability. Not all vaccine clinics can purchase and maintain a solar‐powered television to deliver the vaccine talk. However, alternate ways of providing the vaccine talks, such as audio recorded alone or with a flip chart demonstrating key concepts, have the same general construct and potential for the same effect. We made attempts to standardize the content of the recorded health talk by verifying its content with the Ministry of Health and senior vaccine clinic personnel; however, we did not test its effect on vaccine literacy. This was not the objective of the current study, but determining how to present the most effective, compelling vaccine talk is a topic that should be explored. Finally, beyond the scope of the current study, we did not assess the impact of study intervention on vaccine completion.

## 5 Conclusion

A vaccine clinic workflow change that utilized an audiovisual looped vaccine health education talk over the standard approach significantly reduced vaccine clinic wait time, was feasible to implement and was acceptable to providers and clients. It will be important to study the effect of this mode of health education on vaccine health literacy and vaccine uptake and completion. Research is also required on alternate vaccine delivery methods that may be less expensive to institute and maintain, and be more generalizable to all vaccine clinics.

## Author Contributions

**Rosena O. Oluwafemi:** conceptualization, methodology, writing – review and editing, investigation, data curation, supervision, project administration. **Bukola Ajayi:** methodology, writing – review and editing, investigation, validation, data curation, supervision, project administration. **Eneida A. Mendonca:** methodology, writing – review and editing, supervision, funding acquisition. **Paul Biondich:** conceptualization, writing – review and editing, methodology, supervision, funding acquisition. **Osayame A. Ekhaguere:** conceptualization, writing – review and editing, methodology, funding acquisition, writing – original draft, investigation, supervision, project administration, formal analysis, validation, resources.

## Disclosure

This manuscript does not contain material published elsewhere.

## Ethics Statement

The Ondo State ethical review board approved the study (Protocol # OSHREC08/2/2021/300).

## Consent

Each surveyed provider gave written informed consent. Each surveyed client gave verbal consent. All authors have read and approved the final version of this manuscript.

## Conflicts of Interest

The authors declare no conflicts of interest.

## Data Availability

The datasets used and analysed during the current study are available from the corresponding author upon reasonable request.
